# Systemic immunomodulating therapies for epidermal necrolysis (Stevens‐Johnson syndrome/toxic epidermal necrolysis): A systematic review and meta‐analysis

**DOI:** 10.1111/ddg.15804

**Published:** 2025-09-04

**Authors:** Ruben Heuer, Maren Paulmann, Maja Mockenhaupt, Alexander Nast

**Affiliations:** ^1^ Division of Evidence‐based Medicine (dEBM) Department of Dermatology Venereology and Allergy Charité ‐ University Medicine Berlin Corporate Member of Free University of Berlin Humboldt University of Berlin and Berlin Institute of Health Berlin Germany; ^2^ Dokumentationszentrum schwerer Hautreaktionen (dZh) Department of Dermatology Medical Center – University of Freiburg Freiburg Germany

**Keywords:** drug reaction, epidermal necrolysis, stevens‐Johnson syndrome, systematic Review, toxic epidermal necrolysis

## Abstract

**Background and Objectives:**

Epidermal necrolysis is a rare but severe cutaneous reaction with high mortality. Limited evidence exists regarding the efficacy of systemic immunomodulatory therapies (SITs). Our systematic review aimed to compare SITs with supportive care or one another.

**Patients and Methods:**

Randomized controlled trials and controlled observational studies (≥ 5 patients of all ages per arm) using the international consensus classification for EN (Bastuji‐Garin, 1993) with no significant baseline differences were included. We searched MEDLINE, Embase, and Cochrane CENTRAL (January 1, 1993–January 22, 2024). Two reviewers independently extracted study and outcome data. A random‐effects meta‐analysis was performed.

**Results:**

The main outcome was mortality. Secondary outcomes included hospital stay duration, time to complete reepithelialization, complications, and sequelae. 43 studies with 58 treatment comparisons were analyzed. Cyclosporine was superior to IVIG regarding mortality (RR 0.18, 95% CI 0.05–0.58), and corticosteroids plus IVIG reduced serious complications compared to corticosteroids alone (sepsis, RR 0.77, 95% CI 0.31–0.77). Compared to supportive care, only etanercept showed a significant mortality benefit (RR 0.32, 95% CI 0.11–0.93; GRADE: imprecision/unclear clinical importance).

**Conclusions:**

There is no clear evidence of the superiority of SITs over supportive care, which remains the primary treatment for EN.

## INTRODUCTION

Epidermal necrolysis (EN; also Stevens‐Johnson syndrome / toxic epidermal necrolysis) is an acute, life‐threatening mucocutaneous reaction with high mortality.^1^, ^2^ EN is characterized by epidermal detachment and erosions of mucous membranes, the severity of which is differentiated according to the extent of the epidermal detachment: Stevens‐Johnson syndrome (SJS; < 10%), toxic epidermal necrolysis (TEN; > 30%), and SJS/TEN‐overlap 10‐30%.^3^ EN is rare, with an overall incidence rate of 0.93 cases per 1 million individuals per year (95% CI 0.86–1.00) in Germany.^2^ As a result of the very low incidence rate and considerable heterogeneity in the patient population, performing and interpreting trials in EN is challenging.

The primary objectives in patient management are to initiate supportive care and determine the most likely cause. Because EN is mainly drug‐induced,^4^, ^5^, ^6^ withdrawal of the potential culprit drug is essential.^7^ In addition to supportive care, various systemic immunomodulating treatments (SITs) are used. Corticosteroids, intravenous immunoglobulins (IVIG), cyclosporine, tumor necrosis factor (TNF)‐α inhibitors (e.g., etanercept, infliximab), or a combination of various SITs are routinely administered in clinical practice.^8^, ^9^, ^10^, ^11^

Previous meta‐analyses did not reveal a significant survival benefit for corticosteroids,^8^, ^9^, ^12^ IVIG,^8^, ^9^, ^12^, ^13^ cyclosporine,^9^, ^12^ or the combination of steroids with IVIG,^12^ compared with supportive care. In addition to comparing an intervention with supportive care, some meta‐analyses use the TEN‐specific severity‐of‐illness score (SCORTEN),^14^ to calculate a standardized mortality ratio (SMR; quotient between observed and SCORTEN‐predicted mortality).^15^, ^16^, ^17^, ^18^ However, head‐to‐head comparisons are much less common.

This systematic review and meta‐analysis was conducted as part of the development of the German evidence‐based guideline for the diagnosis and treatment of EN, including one additional update search following the finalization of the guideline. The objectives of this review are *(1)* to provide a comprehensive overview of proposed SITs and *(2)* to estimate their effects on various outcomes compared to supportive care or other SITs.

## PATIENTS AND METHODS

We followed systematic review methods as recommended by Higgins et al.^19^ For each comparison, our certainty in the reported effect estimate was rated according to guidelines proposed by the *Grading of Recommendations Assessment, Development and Evaluation* (GRADE) working group (for methodological details see online supplementary Table S1).^20^ We reported our results in line with the *Preferred Reporting Items for Systematic Reviews and Meta‐Analyses* (PRISMA) guideline. Before data collection and analysis, a protocol was registered on PROSPERO (registration number: CRD42023423396).

### Search strategy and selection/eligibility criteria

On May 3rd, 2023, we searched MEDLINE and Embase (search terms are provided in online supplementary Table S2) for primary studies of interventions. An update search was run on January 22nd, 2024 (Embase, MEDLINE) and April 5th, 2024 (Cochrane CENTRAL).

To reduce the risk of including studies involving non‐EN patients, the search was limited to publications from January 1993 onward – the month of the seminal disease classification by Bastuji‐Garin et al. – as a clear disease definition had not been established prior to that time.^3^ For the same reason, included studies were required to reference this publication or to apply an analogous disease classification (e.g., RegiSCAR criteria).

We included all randomized, controlled, parallel‐group studies and non‐randomized, controlled, parallel‐group studies with at least five participants per treatment arm, regardless of age. We also included uncontrolled cohort studies involving at least two systemic immunomodulatory therapies with at least five participants per treatment arm.

Studies with insufficient information to extract or calculate a summary effect measure for at least one outcome of interest were excluded, as well as studies with incomplete baseline characteristics that also failed to adjust reported effect estimates accordingly (e.g., via multiple regression analysis). As incomplete reporting, we considered missing information on disease severity (at least one of total body surface area affected [TBSA] or SCORTEN) and comorbidities (at least patient age as a proxy measure). We compared baseline characteristics between each study and excluded studies with clinically meaningful differences, operationalized as differences in mean age > 15 years, mean affected body surface area > 15%, and mean expected mortality (according to SCORTEN) > 30%. Exclusion also required statistical significance (*p* < 0.05) of between‐group differences as determined by appropriate hypothesis tests (parametric tests for aggregated effect estimates, non‐parametric tests for studies reporting at the patient level).

No language restriction was applied. To identify potentially missed studies, we conducted backward and forward citation searches by reviewing the reference lists of included articles and publications citing them, as listed on lens.org. Reference management was performed using EndNote.

### Study selection and data extraction

Two reviewers (MP and RH) independently performed study selection and data extraction; disagreements were resolved by discussion. Studies were selected in two steps: *(1)* title and abstract screening and *(2)* evaluation of the full text. In case of missing baseline characteristics and comparative outcomes, individual patient data was used to calculate these measures, if available.

### Data synthesis/analysis

Where possible, we quantitatively synthesized data from individual studies and meta‐analyzed the results if at least two studies reported on the same outcome for the same set of interventions. For dichotomous outcomes (mortality, adverse events), risk ratios were estimated. We calculated unstandardized pooled mean differences for continuous outcomes, since all included studies reported estimates on the same scale (time to complete reepithelialization and length of hospital stay in days). Where only standard errors (SE) or confidence intervals were available for continuous outcomes, we calculated standard deviations via established procedures.^19^ We used R version 4.3.1 (The R Foundation for Statistical Computing Platform) to calculate mean differences (MDs) and risk ratios (RRs) with corresponding 95% CIs. The presented summary measures are based on a random effects model (DerSimonian‐Laird), as study‐specific true effects were assumed due to methodological heterogeneity and historically differential care practices (e.g., supportive care traditions) across countries and treatment centers. Since a fixed effects model using the Mantel‐Haenszel method could also be considered, we have presented its results in the Forest plots and tables to show possible discrepancies between the two approaches.

We carried out the following sensitivity analyses to verify robustness of the pooled estimates: *(1)* varying the cut‐off for clinical significance of baseline between‐group differences (α‐value), *(2)* excluding studies with a high risk of bias, *(3)* excluding studies without deaths in one of the intervention arms, *(4)* varying the method for calculating the τ^2^ estimator.

## RESULTS

### Literature search and characteristics of the included studies

The database search yielded 10,829 references after removal of duplicates. Following the exclusion of unrelated or irretrievable publications, 1,091 articles were assessed in full text. Ultimately, 43 studies met the inclusion criteria and were included in the review (for PRISMA flowchart, see online supplementary Figure S1). Characteristics of all included studies are presented in online supplementary Table S3. Overall, studies from 17 countries – including two randomized controlled trials (RCTs) and 42 observational cohort studies (4 prospective, 38 retrospective, including a supplementary historical comparison from one of the randomized controlled trials) – yielded 21 direct pairwise comparisons. Only two studies reported exclusively on the treatment in children,^21^, ^22^ while the remaining ones focused on adults or included patients of any age group. All studies describe at least two different treatment arms, and the patients were either treated only supportively (21), with corticosteroids (26), IVIG (14), cyclosporine (8), other SITs as monotherapy (4), or with a combination of two SITs (19).

**FIGURE 1:**
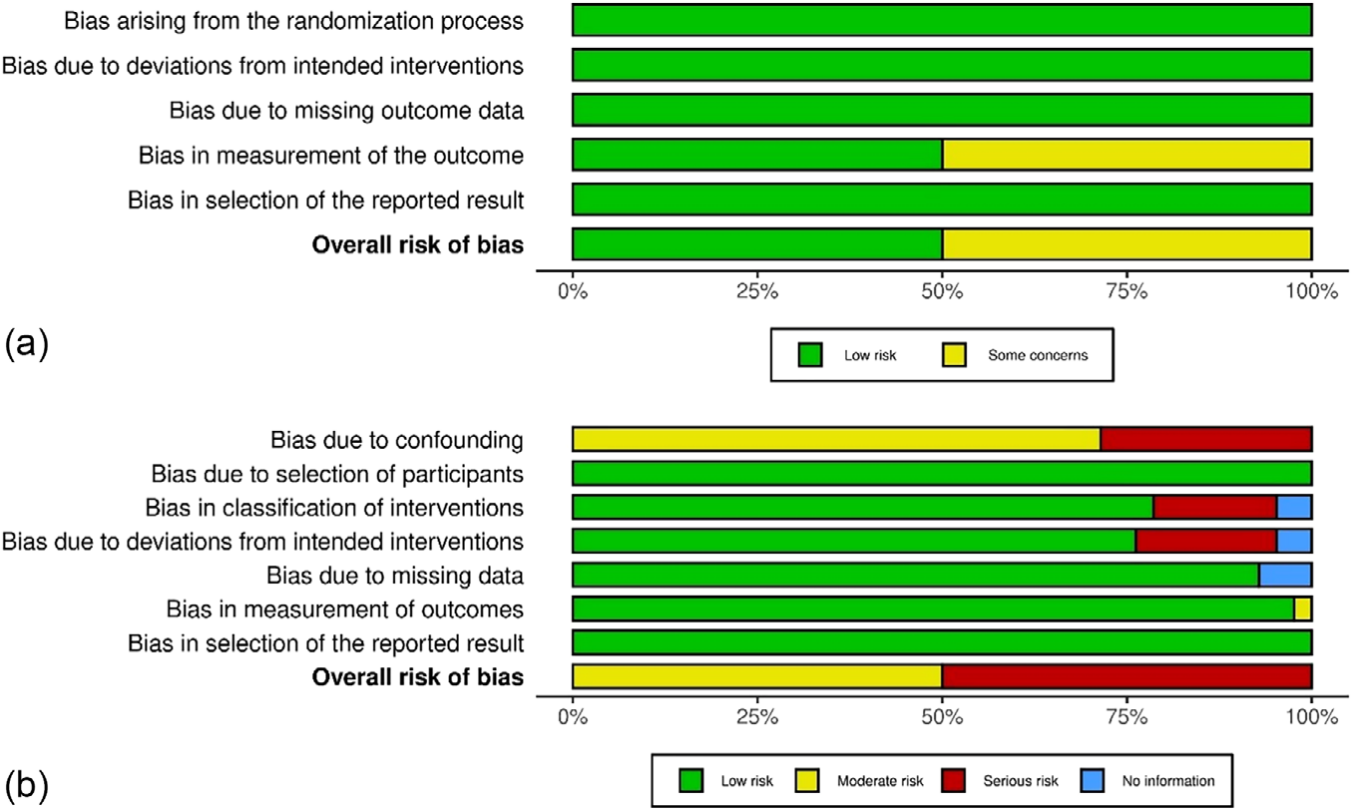
Risk of bias summary. The plots were created with robvis.⁷⁹ (a) Assessment of randomized controlled trials using the Cochrane Risk of Bias tool 2.0 (b) Assessment of non‐randomized studies using ROBINS‐I.

Risk of bias was assessed for all outcomes and presented separately for RCTs *(a)* in Figure 1 (detailed evaluation in online supplementary Figure S2) and for non‐randomized studies *(b)* in Figure 1 (detailed evaluation in online supplementary Figure S3). We used the RoB 2 tool to assess the two RCTs, one of which was evaluated at *low risk of bias*,^23^ and one as leaving *some concerns* related to the measurement of outcomes.^24^ Using the ROBINS‐I tool, we assessed 42 observational studies, half of which reached an overall evaluation of either *moderate* or *high*.^21^, ^22^, ^24^, ^25^, ^26^, ^27^, ^28^, ^29^, ^30^, ^31^, ^32^, ^33^, ^34^, ^35^, ^36^, ^37^, ^38^, ^39^, ^40^, ^41^, ^42^, ^43^, ^44^, ^45^, ^46^, ^47^, ^48^, ^49^, ^50^, ^51^, ^52^, ^53^, ^54^, ^55^, ^56^, ^57^, ^58^, ^59^, ^60^, ^61^, ^62^, ^63^

We originally planned to stratify studies based on patient age, but data proved insufficient for subgroup analyses.

### Mortality

**FIGURE 2:**
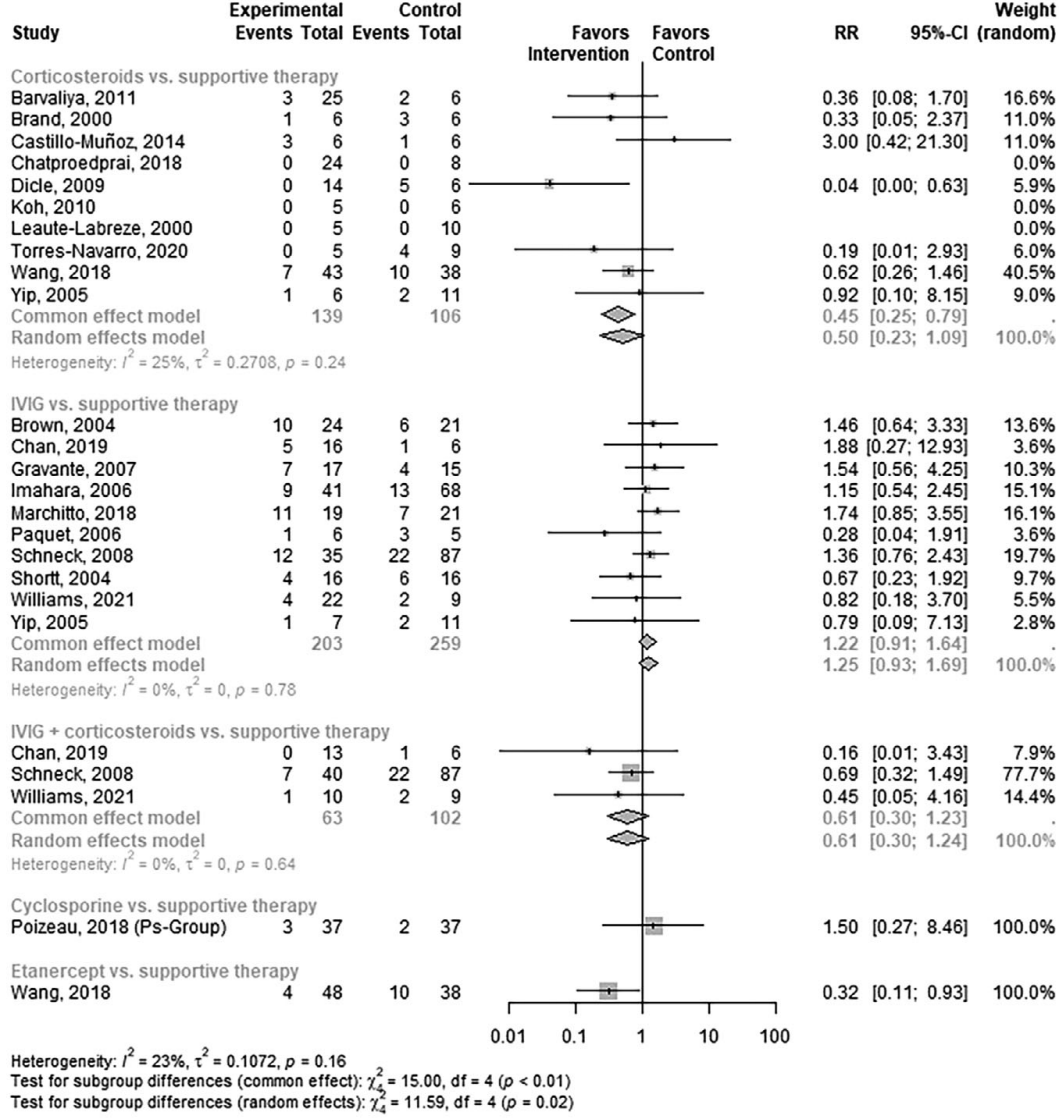
Comparison of systemic immunomodulating treatments and supportive care with respect to mortality.

There were six direct pairwise comparisons for the effect of SITs versus supportive care on mortality (online supplementary Figure S2 and online supplementary Table S4). Figure 2 shows the most commonly used SITs, while other mono‐ or combination therapies can be found in the supplement. Using a random effects model, no statistically significant treatment effect could be demonstrated for corticosteroids (RR 0.5, 95% CI 0.23–1.09). A comparison of etanercept with supportive care resulted in a statistically significant advantage for the intervention (RR 0.32, 95% CI 0.11–0.93; GRADE: imprecision due to wide confidence intervals/unclear clinical importance). The other SITs did not show a statistically significant effect compared to supportive care.

**FIGURE 3:**
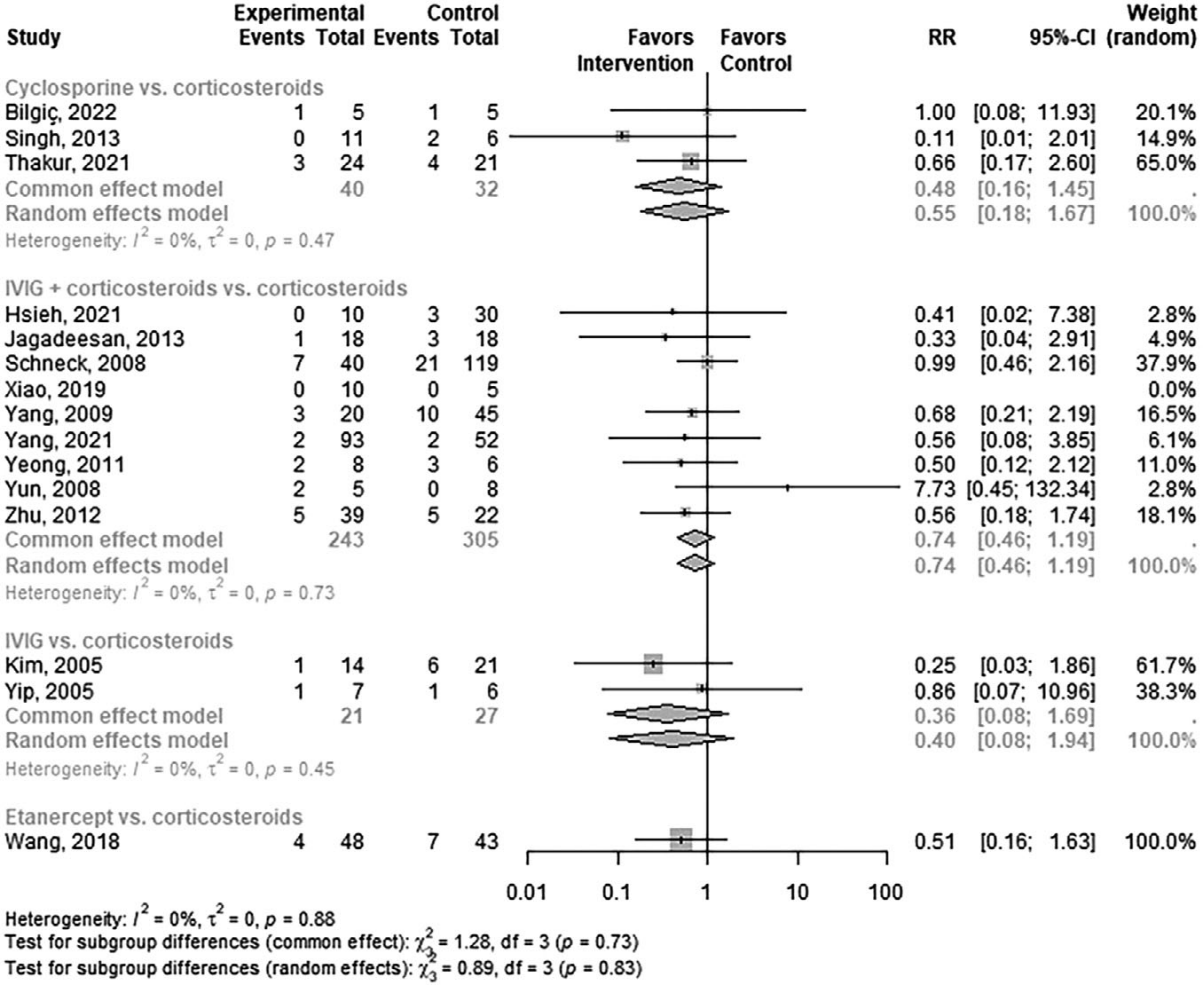
Comparison of systemic immunomodulating treatments and corticosteroids with respect to mortality.

There were seven direct pairwise comparisons for SITs versus corticosteroids (Figure 3 and online supplementary Table S4). Within this comparison group, no intervention demonstrated a statistically significant difference (Figure 3).

In contrast, a statistically significant effect was observed in the following head‐to‐head comparisons: cyclosporine versus IVIG (RR 0.18, 95% CI 0.05–0.58), corticosteroids plus IVIG versus IVIG (RR 0.46, 95% CI 0.22–0.96) and thalidomide in favor of placebo (RR 2.78, 95% CI 1.04–7.4) (online supplementary Table S4).

For each comparison, the number of patients included per treatment arm failed to meet the optimal information size (OIS) criterion. The reported effect estimates must, therefore, be regarded as imprecise.

Visual inspection of the funnel plots and Egger's regression analysis for the only feasible outcome, mortality, revealed no statistical indication of small‐study bias. However, due to the low number of studies per meta‐analysis, only two comparisons could be analyzed. Small study bias can accordingly not be excluded (online supplementary Figure S4).

### Time to complete reepithelialization

A total of nine studies were included to evaluate seven direct pairwise comparisons in terms of time to complete reepithelialization (online supplementary Table S5 and online supplementary Figure S5). The analysis of cyclosporine compared to corticosteroids met the OIS criterion but revealed considerable heterogeneity (*I^2^* > 70%). All three identified studies for this comparison showed an effect in favor of cyclosporine, with two studies demonstrating a significant effect^50^, ^52^ and one without statistical significance.^53^ Compared to corticosteroids plus cyclophosphamide, using cyclosporine resulted in a clinically significantly shorter time to complete reepithelialization (MD –5.7, 95% CI –9–(–2.4)) without meeting the OIS criterion.

Compared to corticosteroids alone, the time to complete reepithelialization was shortened in the combination of corticosteroids plus IVIG (MD –2.93, 95% CI –4.4–(–1.46; GRADE: imprecision due to wide confidence intervals/unclear clinical importance)) and the combination of corticosteroids plus adalimumab (MD –3.5, 95% CI –5.17–(–1.83); GRADE: imprecision due to wide confidence intervals/unclear clinical importance). For all other statistically significant comparisons, confidence intervals crossed the clinical decision threshold, questioning their clinical relevance.

### Length of hospital stay

A total of 21 studies were considered to evaluate nine direct pairwise comparisons on the length of hospital stay (online supplementary Table S6 and online supplementary Figure S6). In five direct pairwise comparisons, only one comparison showed a statistically significant difference (corticosteroids plus IVIG versus corticosteroids) based on studies with considerable heterogeneity (*I^2^* > 70%). The four studies included in this comparison showed an effect in favor of the combination therapy, with two studies demonstrating a significant effect,^56^, ^57^ whereas two did not.^40^, ^55^ Other comparisons with IVIG, either alone or in combination with N‐acetylcysteine or the combination of corticosteroid plus cyclosporine, showed no statistically significant difference.

For each comparison, the number of included patients per treatment arm failed to meet the OIS criterion, rendering the reported effect estimates imprecise.

### Serious complications and sequelae

Various studies were identified and evaluated for the serious complications sepsis, organ failure, and mechanical ventilation (online supplementary Table S7). A total of 15 studies were included to evaluate eight direct pairwise comparisons for sepsis, nine studies of five comparisons for organ failure, and six studies of six comparisons for ventilation. Only corticosteroids compared to supportive care showed a statistically significant effect (serious complications: organ failure, RR 0.27, 95% CI 0.08–0.89). One study could be identified for extraction of skin sequelae, while eight studies of five direct pairwise comparisons of eye sequelae were available (online supplementary Table S8).

No comparison met the OIS criterion.

## DISCUSSION

The literature lists various systemic immunomodulating therapies for EN, but none have shown an unequivocal benefit. Our meta‐analysis included 43 studies with 58 treatment comparisons. In addition to supportive care, the most common interventions were corticosteroids, IVIG, a combination of corticosteroids plus IVIG, and cyclosporine. We analyzed the efficacy of interventions in terms of mortality, time to complete reepithelialization, length of hospital stay, occurrence of serious complications, and long‐term sequelae.

Corticosteroids are widely used to treat EN and represent the most common SIT in Germany.^49^, ^64^ To date, however, there is no randomized controlled trial comparing corticosteroids to supportive care. Existing cohort studies (mostly retrospective single‐arm studies and case reports/series) have shown varying results. Therefore, our results reflect the unresolved debate about corticosteroids. Previous systematic reviews and meta‐analyses failed to establish a statistically significant advantage of corticosteroids over supportive care in terms of mortality; only Zimmermann et al. showed significant results in a secondary analysis using an unstratified model based on individual patient data (OR 0.67, 95% CI 0.46–0.97), whereas their primary analysis on the study level did not show statistically significant results (OR 0.54, 95% CI 0.29–1.01).^8^, ^9^, ^15^

Earlier studies frequently discussed the initiation time of corticosteroids, dosage, and duration of treatment and their effects on mortality, infections, and length of hospital stay.^65^, ^66^, ^67^, ^68^ In our meta‐analysis, we decided not to differentiate between corticosteroid doses and treatment durations due to incomplete data in several studies, which would have strongly limited any further analysis.

Our investigation of IVIG for the treatment of EN did not reveal a favorable effect compared to supportive care in terms of mortality, time to complete reepithelialization, length of hospital stay, and severe complications (sepsis, ventilation). Moreover, no benefit of IVIG was observed compared to other SITs (especially cyclosporine and the combination of corticosteroids plus IVIG). Previous meta‐analyses had already failed to detect favorable effects, particularly regarding mortality.^8^, ^9^, ^12^, ^16^

Due to the different doses and treatment protocols, analyzing the efficacy of IVIG is complex, and the debate on the topic is ongoing. Some studies found a benefit in mortality with high total doses of IVIG (≥ 2 g/kg bodyweight),^69^, ^70^ while others observed higher‐than‐expected mortality.^71^ Analysis of low versus high doses was not possible in our meta‐analysis since only an insufficient number of studies specified the dose.

In contrast to the lack of proof for the efficacy of monotherapies compared to supportive care, in our analysis, the combination of corticosteroids plus IVIG demonstrated a statistically significant benefit over IVIG alone for mortality and over corticosteroids for time to complete reepithelialization. However, previous meta‐analyses failed to establish the benefits of the combination of corticosteroids plus IVIG via statistical significance tests.^9^, ^12^, ^15^, ^72^ As with monotherapies, there are no standard protocols for this combination therapy, resulting in substantial variability of treatment methods.

Various meta‐analyses report lower mortality with cyclosporine compared to supportive care, representing cyclosporine as a promising SIT.^8^, ^9^, ^15^, ^18^, ^73^ In our analysis, only one study was included in this comparison that showed a positive effect on the time to complete reepithelialization.

Contrary to the missing evidence for the efficacy of both IVIG and cyclosporine compared to supportive care concerning mortality, the head‐to‐head comparison of cyclosporine versus IVIG showed a statistically significant difference in favor of cyclosporine. However, this finding needs to be interpreted with caution without established superiority of cyclosporine versus supportive care alone.

In reaction to an increased expression of TNF‐α in samples from affected patients,^74^, ^75^, ^76^ TNF‐α inhibitors are used to treat EN,^23^, ^24^, ^25^, ^34^, ^35^, ^46^, ^61^ although the pathogenetic implications are not yet clarified.^77^ An RCT with thalidomide, which was also included in our meta‐analysis, involved a total of 22 patients with EN, but was terminated prematurely due to increased mortality in the thalidomide group, which was paradoxically correlated with elevated TNF‐α levels.^23^ Based on small samples below the OIS threshold, the two other TNF‐α inhibitors included in our analysis, etanercept, and adalimumab, showed statistically significant treatment effects.^24^, ^35^ Existing meta‐analyses report positive treatment effects for TNF‐α inhibitors, which are, however, based on case reports, case series, and/or one single RCT comparing corticosteroids and etanercept.^9^, ^12^, ^16^, ^78^

### Limitations

Clinical trial data are scarce in EN. The primary research is generally of low quality with respect to methodological rigor and reporting standards. Decisions about which studies to consider in meta‐analyses are therefore not only driven by a desire not to compromise data quality but reflect a balancing act between data quality and statistical power. In non‐randomized studies of interventions (NRSI), confounding presents a major source of bias and requires careful consideration of factors that influence both treatment allocation and the outcome of interest. We opted for considering NRSI but included only studies at minimal risk of confounding. This was addressed by excluding single‐arm studies, which reflect not only treatment effects related to the intervention in question but also center‐specific effects, such as those of different supportive care practices. We excluded studies with clinically meaningful baseline differences in treatment and outcome‐relevant patient characteristics. As the ability to detect these differences depends on sample size and, ultimately, arbitrary cut‐off values for clinical importance, we might have included studies with undetected residual confounding. On the other hand, single‐arm studies that provide detailed information on potential confounders (e.g., SCORTEN scores and supportive care) might have been excluded despite their capacity to integrate with other NRSI studies, leading to a loss of statistical power.

## CONCLUSIONS

Based on our meta‐analysis of published studies, evidence for the superiority of any of the routinely used systemic immunomodulatory treatments over supportive care remains elusive. Our review highlights the importance of high‐quality research data in treating orphan diseases like EN. None of the treatment options revealed a clear superiority over others when considering mortality, time to reepithelialization, or length of hospital stay. But even if systemic treatments are ultimately efficacious, detecting small but true treatment effects in meta‐analyses of NRSI is rendered almost impossible by a lack of research subjects, poor reporting of baseline characteristics and treatment outcomes, diverse study populations, and concomitant therapies. This requires concerted efforts in designing large multicenter studies that meet higher methodological and reporting standards.

## FUNDING AND ACKNOWLEDGMENTS

**Funding/Support**: This research was funded through the Federal Joint Committee (G‐BA) of the joint self‐government of physicians, dentists, hospitals, and health insurance funds in Germany under the funding code 01VSF21002.

**Role of the Funder/Sponsor**: The funding source had no role in the design and conduct of the study; collection, management, analysis, and interpretation of the data; preparation, review, or approval of the manuscript; and the decision to submit the manuscript for publication.

## CONFLICT OF INTEREST STATEMENT

M.M. reported serving on a scientific advisory board for Boehringer Ingelheim, serving as a consultant for Pfizer, receiving honoraria; receiving payment (to the University of Freiburg) from several pharmaceutical companies (including Biogen and Janssen) for the provision of drug safety information; receiving payment for continuing medical education work related to adverse drug reactions from Dermatologische Fortbildungs‐Gesellschaft mbH, Galderma, and RG‐Fortbildungsveranstaltungen. R.H., M.P., and A.N. declare no potential conflicts of interest involving the work under consideration for publication.

## Supporting information



Supplementary information
