# Supplementary material for: Systemic immunomodulating therapies for epidermal necrolysis (Stevens‐Johnson syndrome/toxic epidermal necrolysis): A systematic review and meta‐analysis
Source: J Dtsch Dermatol Ges. 2025 Sep 4;24(1):34–42. doi: 10.1111/ddg.15804 (PMC12800890; doi:10.1111/ddg.15804)
Supplement: Supplementary file 1 — Supplementary information [file DDG-24-34-s001.docx]

# Supplemental online content

**Table S1.** Certainty of evidence and risk of bias assessment

**Table S2.** Search strategy for primary studies

**Table S3.** Overview of included studies

**Table S4.** Mortality

**Table S5.** Time to complete reepithelialization

**Table S6.** Length of hospital stay

**Table S7.** Serious complications

**Table S8.** Sequelae

**Figure S1.** PRISMA flowchart of the selection of studies

**Figure S2**: Risk of bias assessment for randomized controlled trials with RoB 2 tool

**Figure S3**: Risk of bias assessment for non-randomized studies with ROBINS-I tool

**Figure S4:** Funnel plots - mortality

**Figure S5:** Forest plots – time to complete reepithelialization

**Figure S6:** Forest plots – length of hospital stay

**References**

**Table S1.** Certainty of evidence and risk of bias assessment

To quantify our overall level of certainty (GRADE assessment), we separately evaluated each outcome regarding risk of bias, inconsistency, indirectness, imprecision, and publication bias [1]. These categories were addressed as follows:

| Risk of bias | Certainty of evidence was downgraded when one or more risk of bias category was rated as high or several categories were rated as unclear. For this step, we used the Cochrane Risk of Bias tool 2.0 (https://www.riskofbias.info/welcome/rob-2-0-tool) for randomized controlled trials (RCTs) and ROBINS-I (https://www.bmj.com/content/355/bmj.i4919) for non-randomized studies of interventions (NRSI). Each outcome was assessed separately, but no difference between outcomes could be identified within any study. Accordingly, we report our risk of bias assessment at the study level. |
| --- | --- |
| Inconsistency | Downgraded if *I^2^* was larger than 70%. |
| Indirectness | Downgraded in case of deviations from the expected treatment regimen (e.g., sequential as opposed to simultaneous administration of corticosteroids and IVIG to assess their combination). |
| Imprecision | If the confidence intervals of a given effect estimate crossed the cut-off for clinically important difference, disallowing a clear distinction between clinically meaningful and non-meaningful effects, the comparison was regarded as of unclear clinical importance. Cut-off values for clinically important differences for dichotomous outcomes were prespecified as representing 20% benefit or 25% harm (RR <0.8 or >1.25; modified from Guyatt et al.) [2, 3]. We chose a modified anchor-based cut-off value of >2 mean difference (MD) in days for continuous outcomes [4]. An optimal information size criterion (OIS) was established based on the size of a single sufficiently powered study as suggested by GRADE guidelines [2]. For dichotomous outcomes, assuming an event rate of 30%, an α-value of 0.05, a β-value of 0.2 and the aforementioned cut-off values for clinical importance, the OIS was set at 859 (benefit) and 623 (harm) patients per treatment arm. For continuous outcomes, assuming a standard deviation of 5 (time to complete reepithelialization) and 10 (length of hospital stay) suggested an OIS of 99 and 393, respectively. Comparisons failing to meet this criterion were regarded as imprecise. |
| Publication bias | Downgraded in case of visual signs of small study bias (funnel plot inspection) and statistical evidence via Egger’s test. At least eight studies had to be included for any meta-analysis. Otherwise, the GRADE option ‘undetected’ was chosen. |

**Table S2.** Search strategy for primary studies

| **Database** | **Final syntax** |
| --- | --- |
| MEDLINE  Embase | 1 "toxic epidermal necrolys*".ti,ab,kw.  2 (toxic and epidermal and necrolys*).ti,ab,kw.  3 "steven* johnson syndrom*".mp.  4 "Steven*-Johnson-Syndrom*".ti,ab,kw.  5 "Steven* Johnson*".ti,ab,kw.  6 "Stevens-Johnson-Syndrom".ti,ab,kw.  7 "Steven*Johnson*Syndrom*".ti,ab,kw.  8 Lyell*.ti,ab,kw.  9 SJS.ti,ab,kw.  10 (epiderm* adj10 necro*).tw.  11 exp Stevens-Johnson Syndrome/  12 or/1-11  13 systemic immun* therap$.mp.  14 STEROIDS/  15 (steroid$ or corticosteroid$).mp.  16 Adrenal Cortex Hormones/  17 corticoid$.mp.  18 Glucocorticoids/  19 (glucocorticosteroid$ or glucocorticoid$).mp.  20 dexamethasone.mp. or DEXAMETHASONE/  21 prednisolone.mp. or PREDNISOLONE/  22 METHYLPREDNISOLONE/ or methylprednisolone.mp.  23 C#closporin*.mp. or CYCLOSPORINE/  24 Immunoglobulins/  25 (immunoglobulin$ or IVIG).mp.  26 etanercept.mp. or ETANERCEPT/  27 Enbrel.mp.  28 Tumor Necrosis Factor-alpha/  29 anti-tumo?r necrosis factor$.mp.  30 anti-tnf.mp.  31 TNF-alpha inhibitor$.mp.  32 anti-interleukin$.mp.  33 infliximab.mp. or INFLIXIMAB/  34 remicade.mp.  35 exp PLATELETPHERESIS/  36 Plateletpheres$.mp.  37 (platelet and rich and pheres$).mp.  38 PLASMAPHERESIS/  39 plasmapheres$.mp.  40 THALIDOMIDE/  41 Thalidomid$.mp.  42 ACETYLCYSTEINE/  43 Acetylcystein$.mp.  44 N?acetylcystein$.mp.  45 NAC.mp.  46 cyclophosphamide.mp. or CYCLOPHOSPHAMIDE/  47 granulocyte stimulating factor$.mp.  48 hemoperfusion.mp. or HEMOPERFUSION/  49 Azathioprine/  50 azathiop*.mp.  51 Adalimumab/  52 Adalimumab.mp.  53 Golimumab.mp.  54 Certolizumab Pegol/  55 Certolizumab.mp.  56 debrid*.mp.  57 Anti-Infective Agents, Local/  58 Silver Nitrate/  59 (silver* adj3 nitr?t*).mp.  60 Silver Sulfadiazine/  61 (silver* adj3 sulfadiazin*).mp  62 aquacel*.mp.  63 flamazine*.mp. and dressing*.mp.  64 (skin* adj3 allograft*).mp.  65 (skin* adj3 xenograft*).mp.  66 skin* coverage*.mp.  67 biobrane*.mp.  68 suprathel*.mp.  69 epigard*.mp.  70 Epicite.mp.  71 Skin, Artificial/  72 Skin Transplantation/  73 biological dressing.mp.  74 Amnion/  75 Amnio* membran*.mp.  76 skin transplantation.mp.  77 debrid*.mp.  78 Anti-Infective Agents, Local/  79 Silver Nitrate/  80 (silver* adj3 nitr?t*).mp.  81 Lubricant Eye Drops.mp.  82 Restasis.mp.  83 Ikervis.mp.  84 Anti-bacterial agents/ and ophthalmic solutions/tu  85 Symblephar*.mp.  86 Ocular shell*.mp.  87 exp serum/  88 (autologous adj2 serum$).tw.  89 Eyelid Diseases/  90 Tretinoin/  91 Vagina/ and Dilatation/  92 Psychotherapy/  93 Anti-bacterial agents/  94 or/13-93  95 12 and 94  96 95 and 1993:current.(sa_year). |
| Cochrane CENTRAL | 1 toxic NEXT epidermal necrolys*  2 (toxic and epidermal and necrolys*)  3 steven* NEXT johnson NEXT syndrom*  4 Steven* NEXT Johnson-Syndrom*  5 Steven* NEXT Johnson*  6 Stevens-Johnson-Syndrom  7 Steven*Johnson*Syndrom*  8 Lyell*  9 SJS  10 epiderm* adj10 necro*  11 MeSH descriptor: [Stevens-Johnson Syndrome] explode all trees  12 MeSH descriptor: [Stevens-Johnson Syndrome] explode all trees  13 {OR #1-#12} in Trials |

**Table S3.** Overview of included studies

| **Author, Year** | **Study period** | **Study design** | **Country, Setting** | **Comparison** | **No. of patients** | **No. of death** | **Age (SD) in years** | **SJS** | **SJS/TEN-overlap** | **TEN** | **TBSA (SD) in %** | **Pertinent Outcomes** |
| --- | --- | --- | --- | --- | --- | --- | --- | --- | --- | --- | --- | --- |
| Ao, 2022 [5]# | 01/2017- 09/2021 | Retrospective chart review | China,  University hospital | CS + ETN vs. CS | 25 | 0 | 40.2 (18.27) | 17 | 0 | 7 | 19.86 (23.67) | Mortality, time to complete reepithelialization, length of hospital stay, serious complications |
| Arevalo, 2000 [6] | 10/1995-01/1999 | Retrospective case series | Spain,  University hospital | CsA vs. CS + CP | 17 | 3 | 47.32 (18.17) | 0 | 0 | 17 | 81.19 (16.93) | Mortality, time to complete reepithelialization, length of hospital stay |
| Barvaliya, 2011 [7]# | 2006-2009 | Retrospective chart review | India,  University hospital | CS vs. SC | 31 | 5 | 32.7 (15.46) | 11 | 0 | 15 | 29.85 (20.69) | Mortality |
| Bilgiç, 2022 [8] | 01/2015-12/2020 | Retrospective chart review | Turkey, University hospital | CS + CsA vs. CS | 11 | 3 | 41.25 (24.78) | 4 | 2 | 5 | NA (NA) | Mortality, length of hospital stay |
|  |  |  |  | CS + CsA vs. CsA | 11 | 3 | 50.07 (28.58) | 4 | 1 | 6 | NA (NA) |  |
|  |  |  |  | CsA vs. CS | 10 | 2 | 47.9 (26.68) | 6 | 1 | 3 | NA (NA) |  |
| Brand, 2000 [9] | 07/1978-06/1998 | Retrospective chart review | Australia, Burn-equivalent care | CS vs. SC | 12 | 4 | 50.65 (15.69) | 0 | 0 | 12 | NA (NA) | Mortality, length of hospital stay |
| Brown, 2004 [10] | 05/1997-09/2002 | Retrospective chart review | USA, Burn-equivalent care | IVIG vs. SC | 45 | 16 | 45.13 (24.84) | NA | NA | NA | 45.55 (24.98) | Mortality, length of hospital stay |
| Castillo-Muñoz, 2014 [11] | 01/1990-12/2013 | Retrospective chart review | Spain, University hospital | CS vs. SC | 12 | 4 | 49.15 (27.64) | 0 | 2 | 10 | NA (NA) | Mortality, length of hospital stay, sequelae |
| Chan, 2019 [12] | 01/2006-12/2016 | Retrospective chart review | Australia, University hospital (burn-equivalent care) | CS + IVIG vs. IVIG | 29 | 5 | NA (NA) | 5 | 5 | 19 | NA (NA) | Mortality, length of hospital stay |
|  |  |  |  | CS + IVIG vs. SC | 19 | 1 | NA (NA) | 7 | 2 | 10 | NA (NA) |  |
|  |  |  |  | IVIG vs. SC | 22 | 6 | NA (NA) | 4 | 5 | 13 | NA (NA) |  |
| Chatproedprai, 2018 [13]# | 1/1997-12/2016 | Retrospective chart review | Thailand, University hospital | CS vs. SC | 32 | 0 | 9.2 (3.96) | 20 | 3 | 9 | NA (NA) | Mortality, length of hospital stay |
| Dicle, 2009 [14] | 2000-2008 | Retrospective chart review | Turkey, University hospital | CS vs. SC | 20 | 5 | 52.75 (17.05) | 3 | 5 | 12 | 43.74 (28.3) | Mortality |
| Dreyer, 2021 [15] | 2013-2016 | Retrospective chart review | USA, University hospital (two centers) | ETN vs. IVIG | 14 | 2 | NA (NA) | 3 | 4 | 7 | 33.48 (26.89) | Mortality |
| Gong, 2023 [16] | 03/2016-05/2022 | Prospective cohort study | China, University hospital | CS + ADA vs. CS | 83 | 0 | 49.97 (19.96) | 47 | 17 | 19 | NA (NA) | Mortality, time to complete reepithelialization |
| Gonzalez-Herrada, 2017 [17] | 2001-2015 | Retrospective chart review | Spain, University hospital (burn-equivalent care) | CsA vs. IVIG | 37 | 7 | 49.38 (18.42) | 5 | 14 | 18 | 36.51 (25.92) | Mortality |
| Gravante, 2007 [18] | 1/1995-12/2005 | Retrospective chart review | Italy, Burn-equivalent care | IVIG vs. SC | 32 | 11 | 44.87 (20.23) | 1 | 8 | 24 | 61.02 (33.8) | Mortality, length of hospital stay |
| Hsieh, 2021 [19] | 01/2000-03/2019 | Retrospective chart review | Japan, University Hospital (two centers) | CS + IVIG vs. CS | 40 | 3 | NA (NA) | NA | NA | NA | NA (NA) | Mortality |
| Imahara, 2006 [20] | 02/1987-03/2004 | Retrospective chart review | USA, University hospital (burn-equivalent care) | IVIG vs. SC | 109 | 22 | 42.41 (24.31) | NA | NA | NA | 55 (27.88) | Mortality |
| Jagadeesan, 2013 [21] | 02/2008-01/2012 | Prospective open-label trial | India, University hospital | CS + IVIG vs. CS | 36 | 4 | 37 (17.47) | NA | NA | NA | 51.16 (12.81) | Mortality, time to complete reepithelialization, length of hospital stay |
| Kim, 2005 [22] | 11/1990-10/2003 | Retrospective chart review | South Korea, University hospital | IVIG vs. CS | 35 | 7 | NA (NA) | 0 | 0 | 35 | NA (NA) | Mortality |
| Kirchhof, 2014 [23] | 2001-2011 | Retrospective chart review | Canada, University hospital (EN referral center, incl. burn unit) | CsA vs. IVIG | 54 | 12 | 54.16 (20.92) | 22 | 18 | 14 | 24.8 (25.11) | Mortality |
| Koh, 2010 [24] | 01/2001-12/2006 | Retrospective chart review | Singapore, Hospital-based care | CS vs. SC | 11 | 0 | 9.45 (3.48) | 10 | 1 | 0 | NA (NA) | Mortality, length of hospital stay, serious complications |
| Léauté-Labrèze, 2000 [25] | NA | Retrospective chart review | France, Hospital-based care | CS vs. SC | 15 | 0 | 8 (3.12) | 15 | 0 | 0 | NA (NA) | Mortality, sequelae |
| Marchitto, 2018 [26] | 1994-2014 | Retrospective chart review | USA, University hospital | IVIG vs. SC | 40 | 18 | NA (NA) | 0 | 0 | 40 | NA (NA) | Mortality |
| Paquet, 2006 [27] | NA | Retrospective chart review | Belgium,  University and Military hospital (burn-equivalent care) | IVIG vs. SC | 11 | 4 | 45.84 (17.99) | 0 | 0 | 11 | 59 (15.9) | Mortality |
| Paquet, 2014 [28] | NA | Prospective cohort study | Belgium, University and Military hospital (burn-equivalent care) | NAC + IFX vs. NAC | 10 | 3 | NA (NA) | NA | NA | NA | NA (NA) | Mortality |
| Pinheiro, 2013 [29] | 01/1999-12/2010 | Retrospective chart review | Portugal,  University hospital (burn-equivalent care) | IVIG + NAC vs. SC | 15 | 6 | 63.47 (25.75) | NA | NA | NA | 48.13 (27.84) | Mortality, length of hospital stay |
| Poizeau, 2018 [30] | 2005-2016 | Retrospective chart review | France, University hospital (EN referral center) | CsA vs. SC | 74 | 5 | NA (NA) | NA | NA | NA | 19 (24.93) | Mortality, serious complications |
| Schneck, 2008 [31] | 04/1997-12/2001 | Retrospective chart review | Germany and France, large multicenter study | CS + IVIG vs. CS | 159 | 28 | NA (NA) | 68 | 63 | 28 | NA (NA) | Mortality |
|  |  |  |  | CS + IVIG vs. IVIG | 75 | 19 | NA (NA) | 20 | 30 | 25 | NA (NA) |  |
|  |  |  |  | CS + IVIG vs. SC | 127 | 29 | NA (NA) | 44 | 49 | 34 | NA (NA) |  |
|  |  |  |  | IVIG vs. SC | 122 | 34 | NA (NA) | 42 | 41 | 39 | NA (NA) |  |
| Shah, 2021 [32] | NA | Retrospective chart review | USA, Hospital-based | CsA vs. CS | 48 | NA | 48.06 (25.51) | 16 | 12 | 20 | 44.6 (27.4) | Time to complete reepithelialization, length of hospital stay, serious complications |
| Shortt, 2004 [33] | 04/1995-12/2002 | Retrospective chart review | Canada, University hospital (burn-equivalent care) | IVIG vs. SC | 32 | 10 | 52.5 (20.18) | NA | NA | NA | 38 (27.78) | Mortality, length of hospital stay, serious complications |
| Singh, 2013 [34] | 07/2011-06/2012 | Prospective, open, pilot, and uncontrolled study | India, University hospital (ICU or burn unit contingent on disease severity) | CsA vs. CS | 17 | 2 | 30.58 (15.16) | 8 | 4 | 5 | 22.98 (17.22) | Mortality (at 1 month), time to complete reepithelialization, length of hospital stay, serious complications, sequelae |
| Thakur, 2021 [35] | 01/2014-12/2018 | Retrospective chart review | India, University hospital | CsA vs. CS | 45 | 7 | 39.03 (17.5) | 7 | 12 | 26 | 27.48 (12.35) | Mortality, time to complete reepithelialization, length of hospital stay, serious complications |
| Torres-Navarro, 2020 [36] | 01/2013-01/2018 | Retrospective chart review | Spain, University hospital (two centers) | CS vs. SC | 14 | 4 | 49.78 (26.91) | 2 | 0 | 12 | NA (NA) | Mortality, length of hospital stay, serious complications |
| Wang, 2018 [37] | 2009-2015 | Randomized controlled trial (open label / unblinded) | Taiwan, University hospital | ETN vs. CS | 91 | 11 | 56.09 (20.81) | 56 | NA | NA | 18.96 (24.72) | Mortality, time to complete reepithelialization |
|  |  | Randomized controlled trial (open label / unblinded) (historical control group) |  | CS vs. SC | 81 | 17 | 58.04 (24.34) | 52 | NA | NA | NA (NA) |  |
|  |  |  |  | ETN vs. SC | 86 | 14 | 54.17 (20.56) | 56 | NA | NA | NA (NA) |  |
| Williams, 2021 [38] | 01/2009-12/2018 | Retrospective chart review | India, University hospital (pediatric ICU) | CS + IVIG vs. IVIG | 32 | 5 | NA (NA) | NA | NA | NA | NA (NA) | Mortality, serious complications, sequelae |
|  |  |  |  | CS + IVIG vs. SC | 19 | 3 | NA (NA) | NA | NA | NA | NA (NA) |  |
|  |  |  |  | IVIG vs. SC | 31 | 6 | NA (NA) | NA | NA | NA | NA (NA) |  |
| Wolkenstein, 1998 [39] | 05/1995-09/1996 | Randomized controlled trial | France, Multicenter study | Thalidomide vs. placebo | 22 | 13 | NA (NA) | 0 | 0 | 22 | NA (NA) | Mortality |
| Xiao, 2019 [40]# | 06/2009-03/2013 | Retrospective observational study | China, University hospital | CS + IVIG vs. CS | 15 | 0 | 34.13 (19.18) | 7 | 2 | 6 | NA (NA) | Mortality, length of hospital stay |
| Yang, 2009 [41] | 01/1993-10/2007 | Retrospective chart review | China, University hospital (ICU) | CS + IVIG vs. CS | 65 | 13 | 45.19 (21.5) | 18 | 4 | 43 | NA (NA) | Mortality, length of hospital stay |
| Yang, 2021 [42] | 01/2008-12/2019 | Retrospective chart review | China, University hospital (ICU) | CS + IVIG vs. CS | 145 | 4 | 47 (18.27) | 122 | 21 | 11 | 9.85 (7.34) | Mortality, length of hospital stay |
| Yeong, 2011 [43] | 01/2000-12/2006 | Retrospective chart review | Taiwan, University hospital (burn-equivalent care) | CS + IVIG vs. CS | 14 | 5 | 57.47 (24.6) | 0 | 0 | 14 | 69.37 (29.3) | Mortality |
| Yip, 2005 [44] | 07/1995-06/2002 | Retrospective chart review | Singapore, University hospital | CS vs. SC | 17 | 3 | 60.89 (19.82) | 0 | 0 | 17 | NA (NA) | Mortality |
|  |  |  |  | IVIG vs. CS | 13 | 2 | 52.69 (17.65) | 0 | 0 | 13 | NA (NA) |  |
|  |  |  |  | IVIG vs. SC | 18 | 3 | 60.36 (20.98) | 0 | 0 | 18 | NA (NA) |  |
| Yun, 2008 [45] | 10/2001-3/2007 | Retrospective chart review | South Korea, University hospital | CS + IVIG vs. CS | 13 | 2 | 52.09 (18.6) | 0 | 0 | 13 | NA (NA) | Mortality |
| Zhang, 2022 [46] | 01/2014-12/2019 | Retrospective chart review | China, Taiwan, multicenter study | CS + ETN vs. CS + IVIG | 46 | 1 | 46.86 (20.7) | 14 | NA | NA | 35.65 (30.33) | Mortality |
| Zhu, 2012 [47] | 01/2000-04/2010 | Retrospective chart review | China, University hospital (ICU) | CS + IVIG vs. CS | 61 | 10 | 47.05 (19.21) | 0 | 0 | 61 | 90.39 (12.08) | Mortality |

ADA: adalimumab, CP: cyclophosphamide, CS: corticosteroids, CsA: cyclosporine A, ETN: etanercept, ICU: intensive care unit, IFX: infliximab, IVIG: intravenous immunoglobulins, NA: not applicable, NAC: N-acetylcysteine, SC: supportive care; #individual patient data received from study authors

**Table S4:** Mortality

| **Comparison** | **No. of studies** | **Experimental group – No. of patients (events)** | **Control group – No. of patients (events)** | **Random effects model RR  [95%-CI]** | **Common effects model RR [95%-CI]** | **GRADE** | ***I^2^*** | **References** |
| --- | --- | --- | --- | --- | --- | --- | --- | --- |
| CS + ADA vs. CS | 1 | 35 (0) | 48 (0) | NA | NA | ⨁⨁◯◯ LOW | NA | [16] |
| CS + CsA vs. CS | 1 | 6 (2) | 5 (1) | 1.67 [0.21-13.43] | 1.67 [0.21-13.43] | ⨁◯◯◯ VERY LOW | NA | [8] |
| CS + CsA vs. CsA | 1 | 6 (2) | 5 (1) | 1.67 [0.21-13.43] | 1.67 [0.21-13.43] | ⨁◯◯◯ VERY LOW | NA | [8] |
| CS + ETN vs. CS | 1 | 15 (0) | 10 (0) | NA | NA | ⨁⨁◯◯ LOW | NA | [5] |
| CS + ETN vs. CS + IVIG | 1 | 25 (0) | 21 (1) | 0.28 [0.01-6.55] | NA | ⨁◯◯◯ VERY LOW | NA | [46] |
| CS + IVIG vs. CS | 9 | 243 (22) | 305 (47) | 0.74 [0.46-1.19] | 0.74 [0.46-1.19] | ⨁◯◯◯ VERY LOW | 0% | ^[19, 21, 31, 40-43, 45, 47]^ |
| CS + IVIG vs. IVIG | 3 | 63 (8) | 73 (21) | 0.46 [0.22-0.96] | 0.4 [0.19-0.85] | ⨁◯◯◯ VERY LOW | 0% | ^[12, 31, 38]^ |
| CS + IVIG vs. SC | 3 | 63 (8) | 102 (25) | 0.61 [0.3-1.24] | 0.61 [0.3-1.24] | ⨁◯◯◯ VERY LOW | 0% | ^[12, 31, 38]^ |
| CS vs. SC | 10 | 139 (15) | 106 (27) | 0.5 [0.23-1.09] | 0.45 [0.25-0.79] | ⨁◯◯◯ VERY LOW | 25% | ^[7, 9, 11, 13, 14, 24, 25, 36, 37, 44]^ |
| CsA vs. CS | 3 | 40 (4) | 32 (7) | 0.55 [0.18-1.67] | 0.48 [0.16-1.45] | ⨁⨁◯◯ LOW | 0% | ^[8, 34, 35]^ |
| CsA vs. CS + CP | 1 | 11 (0) | 6 (3) | 0.08 [0-1.33] | NA | ⨁⨁◯◯ LOW | NA | [6] |
| CsA vs. IVIG | 2 | 43 (3) | 48 (16) | 0.18 [0.05-0.58] | 0.18 [0.05-0.63] | ⨁⨁⨁◯ MODERATE | 0% | [17, 23] |
| CsA vs. SC | 1 | 37 (3) | 37 (2) | 1.5 [0.27-8.46] | 1.5 [0.27-8.46] | ⨁⨁◯◯ LOW | NA | ^[30]^ |
| ETN vs.CS | 1 | 48 (4) | 43 (7) | 0.51 [0.16-1.63] | 0.51 [0.16-1.63] | ⨁⨁⨁◯ MODERATE | NA | ^[37]^ |
| ETN vs. IVIG | 1 | 9 (0) | 5 (2) | 0.12 [0.01-2] | NA | ⨁⨁◯◯ LOW | NA | [15] |
| ETN vs. SC | 1 | 48 (4) | 38 (10) | 0.32 [0.11-0.93] | 0.32 [0.11-0.93] | ⨁◯◯◯ VERY LOW | NA | ^[37]^ |
| IVIG + NAC vs. SC | 1 | 10 (3) | 5 (3) | 0.5 [0.15-1.64] | 0.5 [0.15-1.64] | ⨁◯◯◯ VERY LOW | NA | [29] |
| IVIG vs. CS | 2 | 21 (2) | 27 (7) | 0.4 [0.08-1.94] | 0.36 [0.08-1.69] | ⨁◯◯◯ VERY LOW | 0% | [22, 44] |
| IVIG vs. SC | 10 | 203 (64) | 259 (66) | 1.25 [0.93-1.69] | 1.22 [0.91-1.64] | ⨁◯◯◯ VERY LOW | 0% | ^[10, 12, 18, 20, 26, 27, 31, 33, 38, 44]^ |
| NAC + IFX vs. NAC | 1 | 5 (2) | 5 (1) | 2 [0.26-15.62] | 2 [0.26-15.62] | ⨁◯◯◯ VERY LOW | NA | [28] |
| Thalidomide vs. placebo | 1 | 12 (10) | 10 (3) | 2.78 [1.04-7.4] | 2.78 [1.04-7.4] | ⨁⨁⨁⨁ HIGH | NA | [39] |

ADA: adalimumab, CP: cyclophosphamide, CS: corticosteroids, CsA: cyclosporine A, ETN: etanercept, IFX: infliximab, IVIG: intravenous immunoglobulins, NA: not applicable, NAC: N-acetylcysteine, SC: supportive care

**Table S5.** Time to complete reepithelialization

| **Comparison** | **No. of studies** | **No. of patients** | **Mean±SD in days** | **Random effects model mean difference  [95%-CI] in days** | **Common effects model mean difference [95%-CI] in days** | **GRADE** | ***I^2^*** | **References** |
| --- | --- | --- | --- | --- | --- | --- | --- | --- |
| CS + ADA vs. CS | 1 | 83 | 18±4.97 | -3.5 [-5.17-(-1.83)] | -3.5 [-5.17-(-1.83)] | ⨁⨁◯◯ LOW | NA | [16] |
| CS + ETN vs. CS | 1 | 25 | 16.7±8.5 | -4.57 [-10.38-1.24] | -4.57 [-10.38-1.24] | ⨁⨁◯◯ LOW | NA | [5] |
| CS + IVIG vs. CS | 1 | 36 | 10.93±2.25 | -2.93 [-4.4-(-1.46)] | -2.93 [-4.4-(-1.46)] | ⨁⨁◯◯ LOW | NA | [21] |
| CsA vs. CS | 3 | 110 | 12.46±5.72 | -3.75 [-7.44-(-0.06)] | -2.34 [-3.4-(-1.28)] | ⨁◯◯◯ VERY LOW | **88%*** | [32, 34, 35] |
| CsA vs. CS + CP | 1 | 17 | 17.7±3.14 | -5.7 [-9-(-2.4)] | -5.7 [-9-(-2.4)] | ⨁⨁◯◯ LOW | NA | [6] |
| ETN vs. CS | 1 | 91 | 16±7.2 | -1.7 [-4.48-1.08] | -1.7 [-4.48-1.08] | ⨁⨁⨁◯ MODERATE | NA | [37] |
| IVIG vs. SC | 1 | 45 | 12.4±5.9 | 5.4 [0.57-10.23] | 5.4 [0.57-10.23] | ⨁⨁◯◯ LOW | NA | [10] |

ADA: adalimumab, CP: cyclophosphamide, CS: corticosteroids, CsA: cyclosporine A, ETN: etanercept, IVIG: intravenous immunoglobulins, NA: not applicable, SC: supportive care, * heterogeneity limits interpretation of pooled estimate

**Table S6.** Length of hospital stay

| **Comparison** | **No. of studies** | **No. of patients** | **Mean±SD in days** | **Random effects model mean difference  [95%-CI] in days** | **Common effects model mean difference [95%-CI] in days** | **GRADE** | ***I^2^*** | **References** |
| --- | --- | --- | --- | --- | --- | --- | --- | --- |
| CS + CsA vs. CS | 1 | 11 | 14.8±5.38 | 15.2 [-3.57-33.97] | 15.2 [-3.57-33.97] | ⨁◯◯◯ VERY LOW | NA | [8] |
| CS + CsA vs. CsA | 1 | 11 | 16.5±4.51 | 13.5 [-5.09-32.09] | 13.5 [-5.09-32.09] | ⨁◯◯◯ VERY LOW | NA | [8] |
| CS + ETN vs. CS | 1 | 25 | 17.5±8.57 | -4.9 [-10.75-0.95] | -4.9 [-10.75-0.95] | ⨁⨁◯◯ LOW | NA | [5] |
| CS + IVIG vs. CS | 4 | 261 | 21.6±13.39 | -4.42 [-7.82-(-1.01)] | -3.7 [-5.03-(-2.38)] | ⨁◯◯◯ VERY LOW | **71%*** | ^[21, 40-42]^ |
| CS vs. SC | 5 | 81 | 18.48±21.23 | -1.52 [-6.66-3.62] | -0.25 [-2.61-2.1] | ⨁◯◯◯ VERY LOW | 62% | [9, 11, 13, 24, 36] |
| CsA vs. CS | 4 | 120 | 15.29±11.4 | -2.95 [-8.2-2.31] | -1.4 [-3.42-0.63] | ⨁◯◯◯ VERY LOW | 81% | ^[8, 32, 34, 35]^ |
| CsA vs. IVIG | 1 | 54 | 26.6±28 | -9.8 [-19.63-0.03] | -9.8 [-19.63-0.03] | ⨁⨁◯◯ LOW | NA | [23] |
| IVIG + NAC vs. SC | 1 | 15 | 4.2±3.35 | 3.6 [0.2-7] | 3.6 [0.2-7] | ⨁◯◯◯ VERY LOW | NA | [29] |
| IVIG vs. SC | 3 | 109 | 19.07±22.38 | 3.04 [-1.29-7.37] | 3.04 [-1.29-7.37] | ⨁⨁◯◯ LOW | 0% | [10, 18, 33] |

CS: corticosteroids, CsA: cyclosporine A, ETN: etanercept, IVIG: intravenous immunoglobulins, NA: not applicable, NAC: N-acetylcysteine, SC: supportive care, * heterogeneity limits interpretation of pooled estimate

**Table S7.** Serious complications

| **Comparison** | **No. of studies** | **Experimental group – No. of patients (events)** | **Control group – No. of patients (events)** | **Random effects model RR  [95%-CI]** | **Common effects model RR [95%-CI]** | **GRADE** | ***I^2^*** | **References** |
| --- | --- | --- | --- | --- | --- | --- | --- | --- |
| **Sepsis** | | | | | | | | |
| CS + IVIG vs. CS | 3 | 67 (7) | 73 (9) | 0.77 [0.31-1.93] | 0.77 [0.31-1.93] | ⨁◯◯◯ VERY LOW | 0% | [41, 43, 47] |
| CS + IVIG vs. IVIG | 1 | 10 (5) | 22 (13) | 0.85 [0.42-1.72] | 0.85 [0.42-1.72] | ⨁⨁◯◯ LOW | NA | [38] |
| CS + IVIG vs. SC | 1 | 10 (5) | 9 (5) | 0.9 [0.38-2.11] | 0.9 [0.38-2.11] | ⨁⨁◯◯ LOW | NA | [38] |
| CS vs. SC | 3 | 25 (0) | 18 (3) | NA | NA | ⨁◯◯◯ VERY LOW | NA | [9, 14, 24] |
| CsA vs. CS | 3 | 48 (4) | 62 (8) | 0.62 [0.21-1.8] | 0.47 [0.15-1.46] | ⨁⨁◯◯ LOW | 0% | ^[32, 34, 35]^ |
| CsA vs CS + CP | 1 | 11 (8) | 6 (5) | 0.87 [0.52-1.45] | 0.87 [0.52-1.45] | ⨁⨁◯◯ LOW | NA | [6] |
| CsA vs. SC [PS] | 1 | 37 (11) | 37 (8) | 1.38 [0.62-3.03] | 1.38 [0.62-3.03] | ⨁⨁◯◯ LOW | NA | [30] |
| ETN vs. CS | 1 | 48 (2) | 43 (4) | 0.45 [0.09-2.32] | 0.45 [0.09-2.32] | ⨁⨁⨁◯ MODERATE | NA | [37] |
| IVIG + NAC vs. SC | 1 | 10 (0) | 5 (1) | 0.17 [0.01-3.61] | NA | ⨁◯◯◯ VERY LOW | NA | ^[29]^ |
| IVIG vs. SC | 1 | 22 (13) | 9 (5) | 1.06 [0.54-2.1] | 1.06 [0.54-2.1] | ⨁⨁◯◯ LOW | NA | ^[33, 38]^ |
| NAC + IFX vs. NAC | 1 | 5 (2) | 5 (1) | 2 [0.26-15.62] | 2 [0.26-15.62] | ⨁◯◯◯ VERY LOW | NA | ^[28]^ |
| **Organ failure** | | | | | | | | |
| CS + ETN vs. CS | 1 | 15 (3) | 10 (3) | 0.67 [0.17-2.67] | 0.67 [0.17-2.67] | ⨁⨁◯◯ LOW | NA | [5] |
| CS + IVIG vs. CS | 2 | 47 (8) | 28 (7) | 0.69 [0.28-1.66] | 0.65 [0.27-1.61] | ⨁◯◯◯ VERY LOW | 0% | [43, 47] |
| CS vs. SC | 4 | 30 (2) | 27 (10) | 0.27 [0.08-0.89] | 0.24 [0.07-0.82] | ⨁◯◯◯ VERY LOW | 0% | ^[9, 14, 24, 36]^ |
| CsA vs. CS + CP | 1 | 11 (6) | 6 (5) | 0.65 [0.34-1.25] | 0.65 [0.34-1.25] | ⨁⨁◯◯ LOW | NA | [6] |
| CsA vs. SC [PS] | 1 | 37 (4) | 37 (0) | 9 [0.5-161.37] | NA | ⨁⨁◯◯ LOW | NA | [30] |
| **Ventilation** | | | | | | | | |
| CS + IVIG vs. CS | 1 | 39 (8) | 22 (4) | 1.13 [0.38-3.32] | 1.13 [0.38-3.32] | ⨁⨁◯◯ LOW | NA | [47] |
| CS + IVIG vs. IVIG | 1 | 10 (1) | 22 (5) | 0.44 [0.06-3.29] | 0.44 [0.06-3.29] | ⨁⨁◯◯ LOW | NA | [38] |
| CS + IVIG vs. SC | 1 | 10 (1) | 9 (2) | 0.45 [0.05-4.16] | 0.45 [0.05-4.16] | ⨁⨁◯◯ LOW | NA | [38] |
| CsA vs. CS | 1 | 11 (0) | 6 (1) | 0.19 [0.01-3.99] | NA | ⨁⨁◯◯ LOW | NA | [34] |
| ETN vs. CS | 1 | 48 (3) | 43 (5) | 0.54 [0.14-2.12] | 0.54 [0.14-2.12] | ⨁⨁⨁◯ MODERATE | NA | [37] |
| IVIG vs. SC | 1 | 22 (5) | 9 (2) | 1.02 [0.24-4.34] | 1.02 [0.24-4.34] | ⨁⨁◯◯ LOW | NA | [38] |

CP: cyclophosphamide, CS: corticosteroids, CsA: cyclosporine A, ETN: etanercept, IVIG: intravenous immunoglobulins, NA: not applicable, PS: propensity score-matched, SC: supportive care

**Table S8.** Sequelae

| **Comparison** | **No. of studies** | **Experimental group – No. of patients (events)** | **Control group – No. of patients (events)** | **Random effects model RR  [95%-CI]** | **Common effects model RR [95%-CI]** | ***GRADE*** | ***I^2^*** | **References** |
| --- | --- | --- | --- | --- | --- | --- | --- | --- |
| **skin** | | | | | | | | |
| CS vs. SC | 1 | 5 (2) | 10 (1) | 4 [0.47-34.24] | 4 [0.47-34.24] | ⨁◯◯◯ VERY LOW | NA | [25] |
| **eyes** | | | | | | | | |
| CS + CsA vs. CS | 1 | 6 (1) | 5 (2) | 0.42 [0.05-3.36] | 0.42 [0.05-3.36] | ⨁◯◯◯ VERY LOW | NA | [8] |
| CS + CsA vs. CsA | 1 | 6 (1) | 5 (3) | 0.28 [0.04-1.91] | 0.28 [0.04-1.91] | ⨁◯◯◯ VERY LOW | NA | [8] |
| CS + IVIG vs. IVIG | 1 | 10 (3) | 22 (12) | 0.55 [0.2-1.53] | 0.55 [0.2-1.53] | ⨁⨁◯◯ LOW | NA | [38] |
| CS vs. SC | 3 | 25 (4) | 22 (3) | 1.1 [0.27-4.56] | 1.32 [0.3-5.94] | ⨁◯◯◯ VERY LOW | 0% | ^[11, 14, 25]^ |
| CsA vs. CS | 2 | 16 (4) | 11 (2) | 1.53 [0.47-5.02] | 1.85 [0.48-7.14] | ⨁⨁◯◯ LOW | 0% | ^[8, 34]^ |

CS: corticosteroids, CsA: cyclosporine A, IVIG: intravenous immunoglobulins, NA: not applicable, SC: supportive care

**Figure S1.** PRISMA flowchart of the selection of the primary studies

Reports identified in MEDLINE (2784), Embase (n = 7742), Cochrane CENTRAL (558), and hand search (2443)

**Identification**

Studies identified through database searches (n = 13527)

Duplicate records excluded (n = 2698)

Records screened by title and abstract (n = 10829)

Records excluded in title/abstract screening (n = 9738)

**Screening**

Records screened by full text (n = 1091)

Records not retrieved (n = 31)

Full text articles assessed for eligibility (n = 1060)

Articles excluded (n = 1017)

- Reports with <5 patients or no control group (n = 349)
- No diagnostic certainty (n = 8)
- Baseline characteristics missing or highly different between groups (n = 155)
- Conference abstracts (n = 53)
- Other reasons (e.g., duplicate, review, wrong study design) (n = 452)

**Eligibility**

**Included**

Studies finally included in review (n = 43)

**Figure S2.** Risk of bias assessment for randomized controlled trials with RoB 2 tool


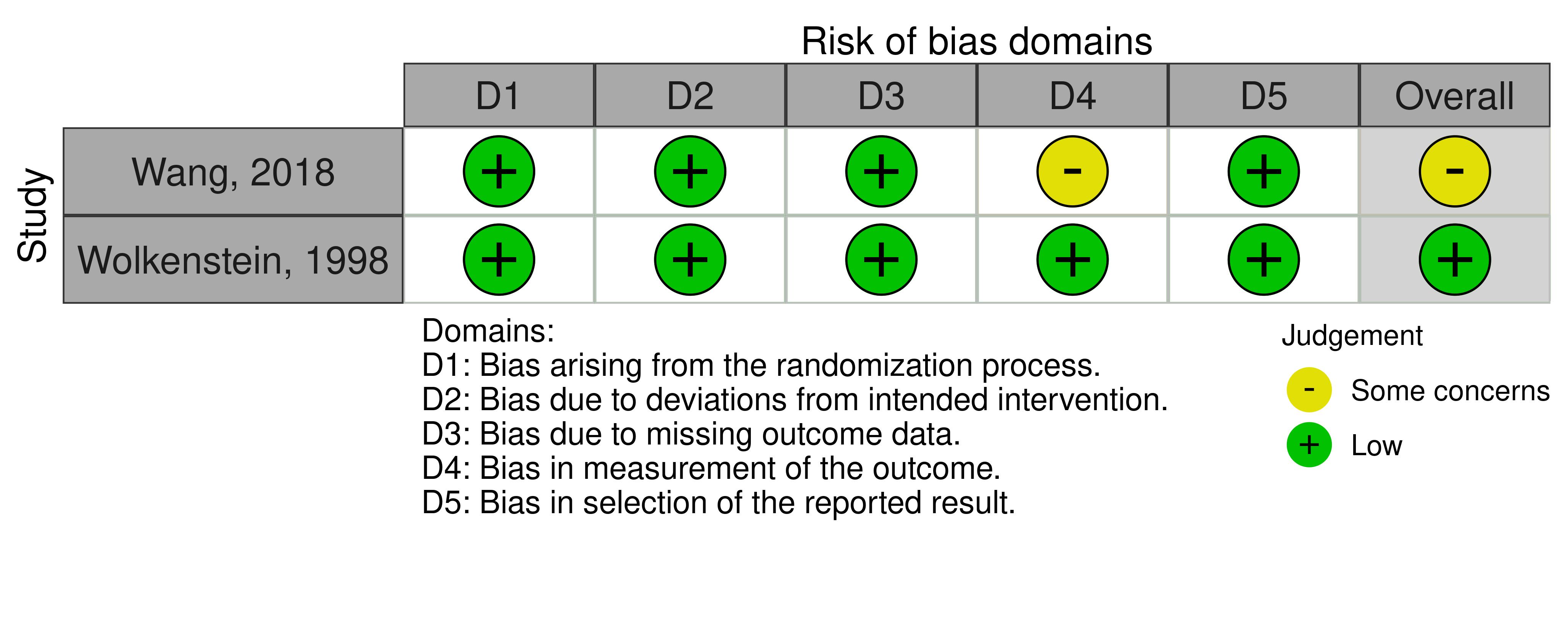


Plot was created with robvis [48].

**Figure S3.** Risk of bias assessment for non-randomized studies with ROBINS-I tool


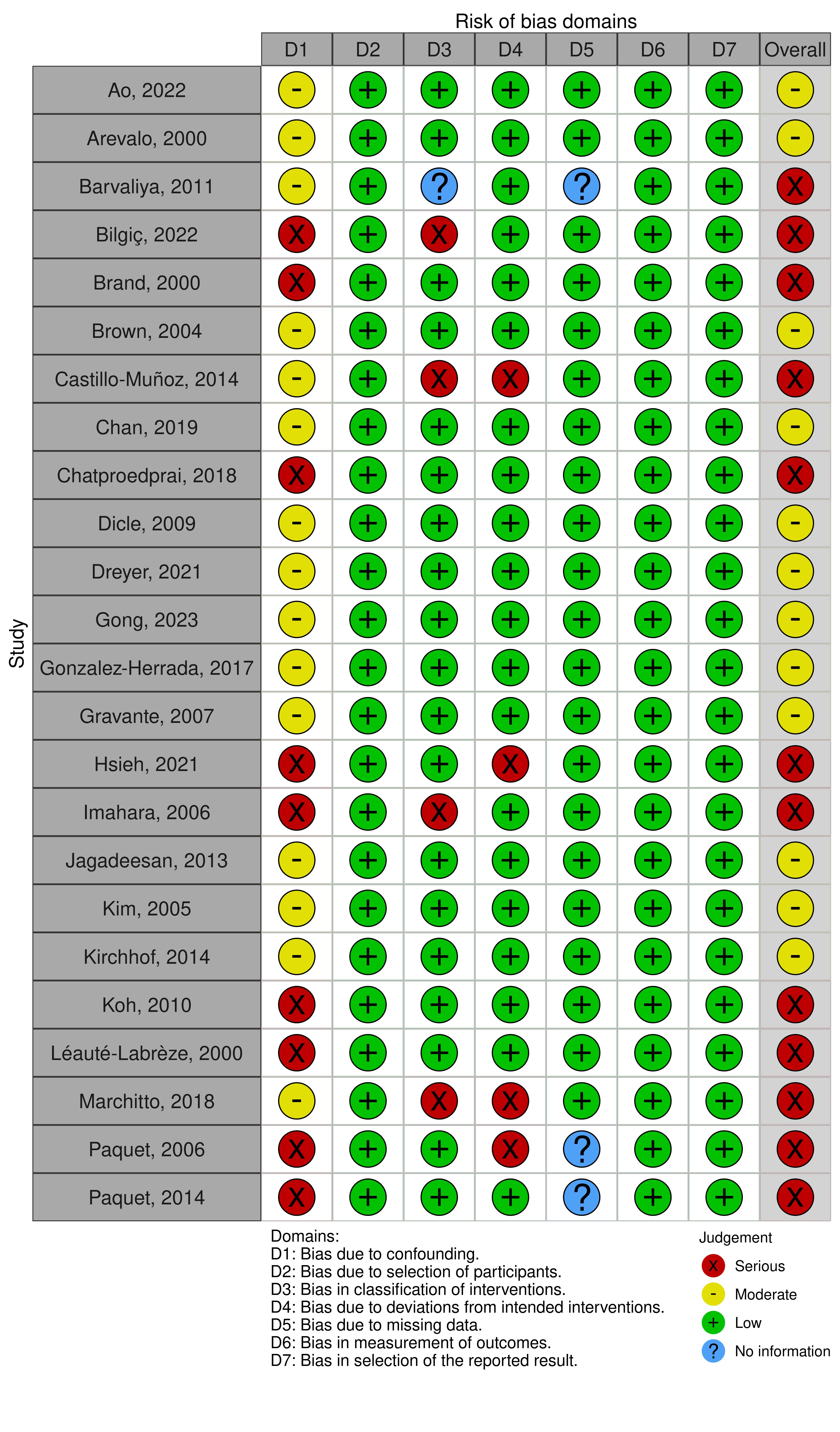

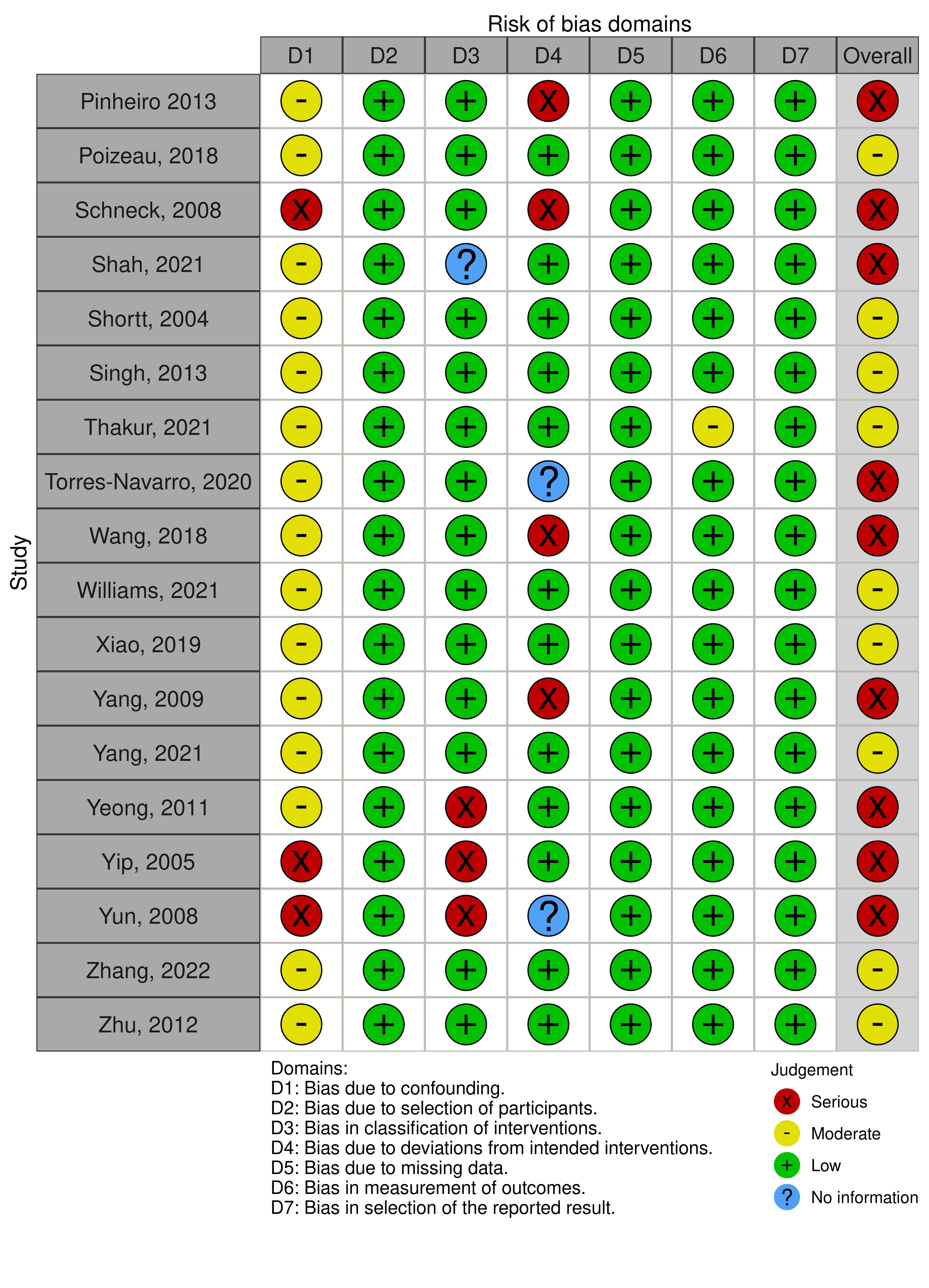


This plot was created with robivs [48].

**Figure S4.** Funnel plots - mortality

(A) Comparison of IVIG plus corticosteroids versus corticosteroids (Egger’s test for funnel plot asymmetry: z = -0.1771, p = 0.8594)


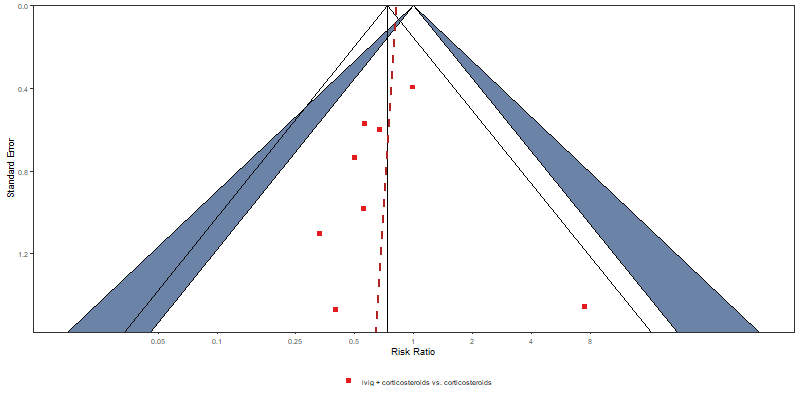


(B) Comparison of IVIG versus supportive therapy (Egger’s test for funnel plot asymmetry:

z = -1.2679, p = 0.2048)


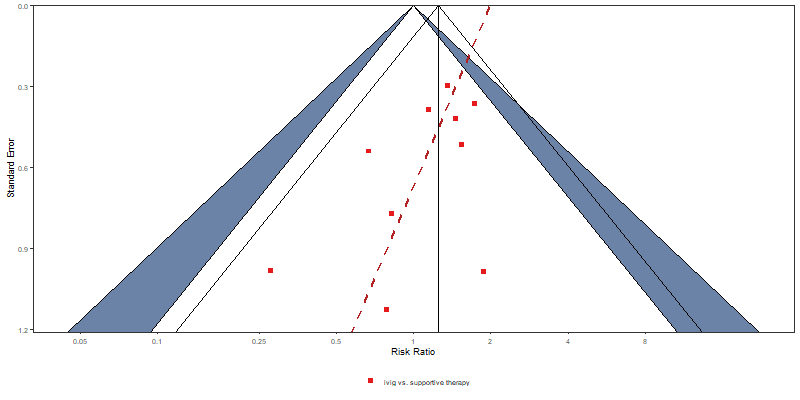


**Figure S5.** Forest plots – time to complete reepithelialization

(A) Comparison of systemic immunomodulating treatment and supportive care


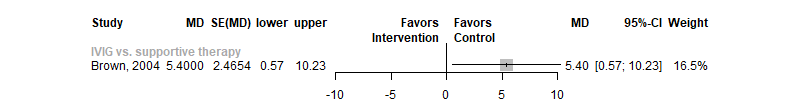


(B) Comparison of different immunomodulating treatments and corticosteroids


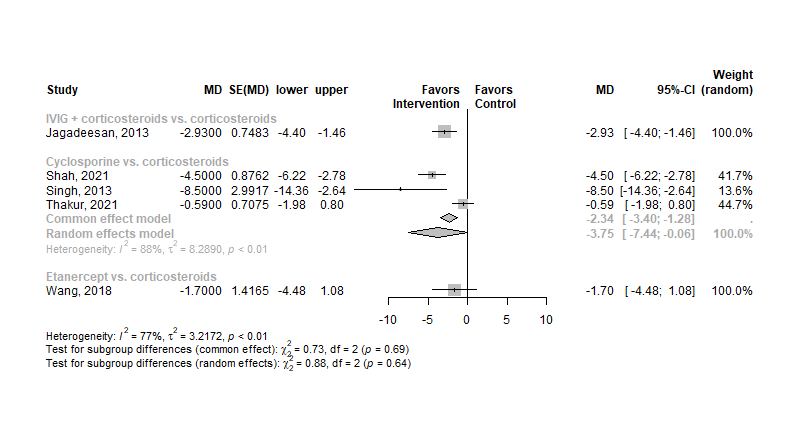


The pooled effect estimate for the comparison cyclosporine A versus corticosteroids is not reported in the results section due to considerable heterogeneity (I^2^ >70%).

**Figure S6.** Forest plots – length of hospital stay

(A) Comparison of different systemic immunomodulating treatments and supportive care

**
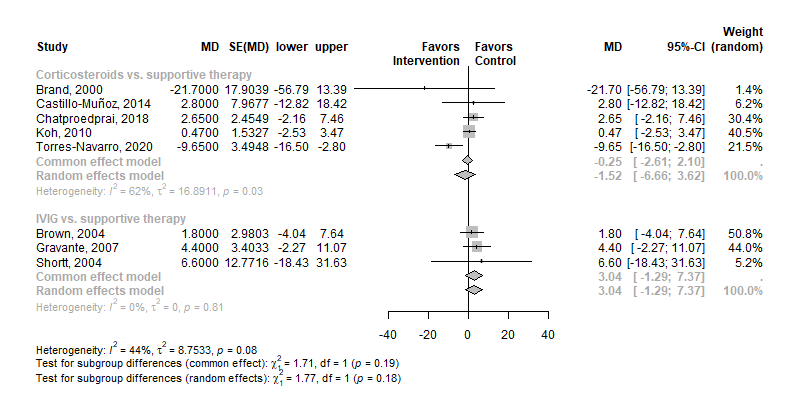
**

(B) Comparison of different systemic immunomodulating treatments and corticosteroids

**
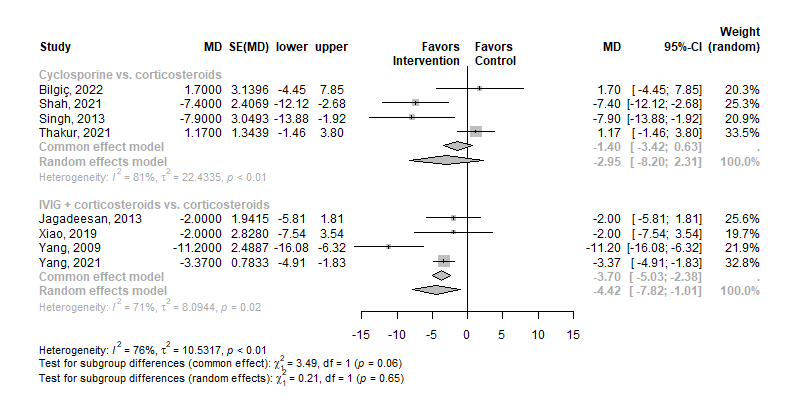
**

The pooled effect estimates for the comparisons cyclosporine versus corticosteroids and corticosteroids plus IVIG versus corticosteroids are not reported in the results section due to considerable heterogeneity (I^2^ >70%).

**References**

1. Guyatt GH, Oxman AD, Schünemann HJ, Tugwell P, Knottnerus A. GRADE guidelines: a new series of articles in the Journal of Clinical Epidemiology. J Clin Epidemiol. 2011; 64: 380-2.

2. Guyatt GH, Oxman AD, Kunz R, Brozek J, Alonso-Coello P, Rind D, Devereaux PJ, Montori VM, Freyschuss B, Vist G, Jaeschke R, Williams JW, Jr., Murad MH, Sinclair D, Falck-Ytter Y, Meerpohl J, Whittington C, Thorlund K, Andrews J, Schünemann HJ. GRADE guidelines 6. Rating the quality of evidence--imprecision. J Clin Epidemiol. 2011; 64: 1283-93.

3. Deutsche Gesellschaft für Koloproktologie e.V. (DGK). Evidenzbericht zur S3-Leitlinie Analkarzinom (Diagnostik, Therapie und Nachsorge von Analkanal und Analrandkarzinomen). AWMF. 2021; AWMF-Registernummer: 081/004OL.

4. Kim WB, Worley B, Holmes J, Phillips EJ, Beecker J. Minimal clinically important differences for measures of treatment efficacy in Stevens-Johnson syndrome and toxic epidermal necrolysis. J Am Acad Dermatol. 2018; 79: 1150-52.

5. Ao S, Gao X, Zhan J, Ai L, Li M, Su H, Tang X, Chu C, Han J, Wang F. Inhibition of tumor necrosis factor improves conventional steroid therapy for Stevens-Johnson syndrome/toxic epidermal necrolysis in a cohort of patients. J Am Acad Dermatol. 2022; 86: 1236-45.

6. Arevalo JM, Lorente JA, Gonzalez-Herrada C, Jimenez-Reyes J. Treatment of toxic epidermal necrolysis with cyclosporin A. J Trauma. 2000; 48: 473-8.

7. Barvaliya M, Sanmukhani J, Patel T, Paliwal N, Shah H, Tripathi C. Drug-induced Stevens-Johnson syndrome (SJS), toxic epidermal necrolysis (TEN), and SJS-TEN overlap: A multicentric retrospective study. J Postgrad Med. 2011; 57: 115-19.

8. Bilgiç A, Mammadli K, İlhan HD, Dursun O, Yılmaz M, Alpsoy E. Retrospective analysis of cases with Stevens-Johnson syndrome/toxic epidermal necrolysis: A case series of 20 patients. Turk J Dermatol. 2022; 16: 80-86.

9. Brand R, Rohr JB. Toxic epidermal necrolysis in Western Australia. Australas J Dermatol. 2000; 41: 31-3.

10. Brown KM, Silver GM, Halerz M, Walaszek P, Sandroni A, Gamelli RL. Toxic epidermal necrolysis: does immunoglobulin make a difference? J Burn Care Rehabil. 2004; 25: 81-8.

11. Castillo-Muñoz FI, Céspedes-Guirao FJ, Novo-Torres A, Lorda-Barraguer E. Análisis retrospectivo de 23 años de necrólisis epidérmica tóxica en la Unidad de Quemados de Alicante, España. Cir Plást Iberolatinoam. 2014; 40: 279-94.

12. Chan L, Cook DK. A 10-year retrospective cohort study of the management of toxic epidermal necrolysis and Stevens-Johnson syndrome in a New South Wales state referral hospital from 2006 to 2016. Int J Dermatol. 2019; 58: 1141-47.

13. Chatproedprai S, Wutticharoenwong V, Tempark T, Wananukul S. Clinical Features and Treatment Outcomes among Children with Stevens-Johnson Syndrome and Toxic Epidermal Necrolysis: A 20-Year Study in a Tertiary Referral Hospital. Dermatol Res Pract. 2018; 2018: 3061084.

14. Dicle Ö, Yılmaz E, Alpsoy E. Stevens-Johnson Syndrome and Toxic Epidermal Necrolysis: A Retrospective Evaluation. [Turkish]. Turkderm-Turk Arch Dermatol Venereol. 2009; 43: 15-20.

15. Dreyer SD, Torres J, Stoddard M, Leavitt E, Sutton A, Aleshin M, Crew A, Worswick S. Efficacy of Etanercept in the Treatment of Stevens-Johnson Syndrome and Toxic Epidermal Necrolysis. Cutis. 2021; 107: E22-E28.

16. Gong T, Zhang P, Ruan S-F, Xiao Z, Chen W, Lin M, Zhong Q, Luo R, Xu Q, Peng J, Cheng B, Chen F, Chen L, Chung W-H, Ji C. APOA4 as a novel predictor of prognosis in Stevens-Johnson syndrome/toxic epidermal necrolysis: A proteomics analysis from two prospective cohorts. J Am Acad Dermatol. 2023; 89: 45-52.

17. González-Herrada C, Rodríguez-Martín S, Cachafeiro L, Lerma V, González O, Lorente JA, Rodríguez-Miguel A, González-Ramos J, Roustan G, Ramírez E, Bellón T, de Abajo FJ, PIELenRed Therapeutic Management Working Group. Cyclosporine Use in Epidermal Necrolysis Is Associated with an Important Mortality Reduction: Evidence from Three Different Approaches. J Invest Dermatol. 2017; 137: 2092-100.

18. Gravante G, Delogu D, Marianetti M, Trombetta M, Esposito G, Montone A. Toxic epidermal necrolysis and Steven Johnson syndrome: 11-years experience and outcome. Eur Rev Med Pharmacol Sci. 2007; 11: 119-27.

19. Hsieh MH, Watanabe T, Aihara M. Recent Dermatological Treatments for Stevens-Johnson Syndrome and Toxic Epidermal Necrolysis in Japan. Front Med (Lausanne). 2021; 8: 636924.

20. Imahara SD, Holmes 4th JH, Heimbach DM, Engrav LE, Honari S, Klein MB, Gibran NS. SCORTEN overestimates mortality in the setting of a standardized treatment protocol. J Burn Care Res. 2006; 27: 270-5.

21. Jagadeesan S, Sobhanakumari K, Sadanandan S, Ravindran S, Divakaran M, Skaria L, Kurien G. Low dose intravenous immunoglobulins and steroids in toxic epidermal necrolysis: A prospective comparative open-labelled study of 36 cases. Indian J Dermatol Venereol Leprol. 2013; 79: 506-11.

22. Kim KJ, Lee DP, Suh HS, Lee MW, Choi JH, Moon KC, Koh JK. Toxic epidermal necrolysis: analysis of clinical course and SCORTEN-based comparison of mortality rate and treatment modalities in Korean patients. Acta Derm Venereol. 2005; 85: 497-502.

23. Kirchhof MG, Miliszewski MA, Sikora S, Papp A, Dutz JP. Retrospective review of Stevens-Johnson syndrome/toxic epidermal necrolysis treatment comparing intravenous immunoglobulin with cyclosporine. J Am Acad Dermatol. 2014; 71: 941-7.

24. Koh MJ, Tay YK. Stevens-Johnson syndrome and toxic epidermal necrolysis in Asian children. J Am Acad Dermatol. 2010; 62: 54-60.

25. Léauté-Labrèze C, Lamireau T, Chawki D, Maleville J, Taieb A. Diagnosis, classification, and management of erythema multiforme and Stevens-Johnson syndrome. Arch Dis Child. 2000; 83: 347-52.

26. Marchitto MC, Sung S, Doong J, Chien AL. Toxic epidermal necrolysis: a review of 20 years of data. J Eur Acad Dermatol Venereol. 2018; 32: e263-e64.

27. Paquet P, Kaveri S, Jacob E, Pirson J, Quatresooz P, Pierard GE. Skin immunoglobulin deposition following intravenous immunoglobulin therapy in toxic epidermal necrolysis. Exp Dermatol. 2006; 15: 381-6.

28. Paquet P, Jennes S, Rousseau AF, Libon F, Delvenne P, Pierard GE. Effect of N-acetylcysteine combined with infliximab on toxic epidermal necrolysis. A proof-of-concept study. Burns. 2014; 40: 1707-12.

29. Pinheiro S, Carvalho R, Ramos S, Diogo C, Caetano M, Cabral L, Cruzeiro C. Toxic epidermal necrolysis: The experience of coimbra's burn unit. Acta Med Port. 2013; 26: 341-48.

30. Poizeau F, Gaudin O, Le Cleach L, Duong TA, Hua C, Hotz C, Ingen-Housz-Oro S, Sbidian E, Zehou O, Colin A, de Prost N, Lebrun-Vignes B, Chosidow O, Wolkenstein P, Fardet L. Cyclosporine for Epidermal Necrolysis: Absence of Beneficial Effect in a Retrospective Cohort of 174 Patients-Exposed/Unexposed and Propensity Score-Matched Analyses. J Invest Dermatol. 2018; 138: 1293-300.

31. Schneck J, Fagot JP, Sekula P, Sassolas B, Roujeau JC, Mockenhaupt M. Effects of treatments on the mortality of Stevens-Johnson syndrome and toxic epidermal necrolysis: A retrospective study on patients included in the prospective EuroSCAR Study. J Am Acad Dermatol. 2008; 58: 33-40.

32. Shah R, Chen ST, Kroshinsky D. Use of cyclosporine for the treatment of Stevens-Johnson syndrome/toxic epidermal necrolysis. J Am Acad Dermatol. 2021; 85: 512-13.

33. Shortt R, Gomez M, Mittman N, Cartotto R. Intravenous immunoglobulin does not improve outcome in toxic epidermal necrolysis. J Burn Care Rehabil. 2004; 25: 246-55.

34. Singh GK, Chatterjee M, Verma R. Cyclosporine in Stevens Johnson syndrome and toxic epidermal necrolysis and retrospective comparison with systemic corticosteroid. Indian J Dermatol Venereol Leprol. 2013; 79: 686-92.

35. Thakur V, Vinay K, Kumar S, Choudhary R, Kumar A, Parsad D, Kumaran MS. Factors Predicting the Outcome of Stevens-Johnson Syndrome and Toxic Epidermal Necrolysis: A 5-Year Retrospective Study. Indian Dermatol Online J. 2021; 12: 258-65.

36. Torres-Navarro I, Briz-Redon A, Botella-Casas G, Sahuquillo-Torralba A, Calle-Andrino A, de Unamuno-Bustos B, Piqueras-Garcia J, Roca Gines J, Magdaleno Tapial J, Alegre de Miquel V, Botella-Estrada R. Accuracy of SCORTEN and ABCD-10 to predict mortality and the influence of renal function in Stevens-Johnson syndrome/toxic epidermal necrolysis. J Dermatol. 2020; 47: 1182-86.

37. Wang CW, Yang LY, Chen CB, Ho HC, Hung SI, Yang CH, Chang CJ, Su SC, Hui RC, Chin SW, Huang LF, Lin YY, Chang WY, Fan WL, Yang CY, Ho JC, Chang YC, Lu CW, Chung WH, the Taiwan Severe Cutaneous Adverse Reaction C. Randomized, controlled trial of TNF-alpha antagonist in CTL-mediated severe cutaneous adverse reactions. J Clin Invest. 2018; 128: 985-96.

38. Williams V, Reddy M, Bansal A, Baranwal AK, Nallasamy K, Angurana SK, Handa S, Ram J, Jayashree M, Singhi S. Intensive care needs and long-term outcome of pediatric toxic epidermal necrolysis - A 10-year experience. Int J Dermatol. 2021; 60: 44-52.

39. Wolkenstein P, Latarjet J, Roujeau JC, Duguet C, Boudeau S, Vaillant L, Maignan M, Schuhmacher MH, Milpied B, Pilorget A, Bocquet H, Brun-Buisson C, Revuz J. Randomised comparison of thalidomide versus placebo in toxic epidermal necrolysis. Lancet. 1998; 352: 1586-9.

40. Xiao Y, Gang W. Clinical analysis of severe drug eruption treated with hemoperfusion and continuous renal replacement therapy combined. Journal of the Dermatology Nurses' Association Conference: 24th World Congress of Dermatology Milan Italy. 2019; 12.

41. Yang Y, Xu J, Li F, Zhu X. Combination therapy of intravenous immunoglobulin and corticosteroid in the treatment of toxic epidermal necrolysis and Stevens-Johnson syndrome: a retrospective comparative study in China. Int J Dermatol. 2009; 48: 1122-8.

42. Yang L, Shou YH, Li F, Zhu XH, Yang YS, Xu JH. Intravenous Immunoglobulin Combined With Corticosteroids for the Treatment of Stevens-Johnson Syndrome/Toxic Epidermal Necrolysis: A Propensity-Matched Retrospective Study in China. Front Pharmacol. 2022; 12: 750173.

43. Yeong EK, Lee CH, Hu FC, M ZW. Serum bicarbonate as a marker to predict mortality in toxic epidermal necrolysis. J Intensive Care Med. 2011; 26: 250-4.

44. Yip LW, Thong BY, Tan AW, Khin LW, Chng HH, Heng WJ. High-dose intravenous immunoglobulin in the treatment of toxic epidermal necrolysis: a study of ocular benefits. Eye. 2005; 19: 846-53.

45. Yun SJ, Choi MS, Piao MS, Lee JB, Kim SJ, Won YH, Lee SC. Serum lactate dehydrogenase is a novel marker for the evaluation of disease severity in the early stage of toxic epidermal necrolysis. Dermatology. 2008; 217: 254-59.

46. Zhang J, Lu CW, Chen CB, Wang CW, Chen WT, Cheng B, Ji C, Chung WH. Evaluation of Combination Therapy With Etanercept and Systemic Corticosteroids for Stevens-Johnson Syndrome and Toxic Epidermal Necrolysis: A Multicenter Observational Study. J Allergy Clin Immunol Pract. 2022; 10: 1295-304.e6.

47. Zhu QY, Ma L, Luo XQ, Huang HY. Toxic epidermal necrolysis: performance of SCORTEN and the score-based comparison of the efficacy of corticosteroid therapy and intravenous immunoglobulin combined therapy in China. J Burn Care Res. 2012; 33: e295-308.

48. McGuinness LA, Higgins JPT. Risk-of-bias VISualization (robvis): An R package and Shiny web app for visualizing risk-of-bias assessments. Res Synth Methods. 2021; 12: 55-61.
